# Primary squamous cell carcinoma of endometrium: case report and literature review

**DOI:** 10.11604/pamj.2018.30.208.9654

**Published:** 2018-07-16

**Authors:** Fatima Zahra Farhane, Zineb Alami, Touria Bouhafa, Abderrahmane Elmazghi, Khalid Hassouni

**Affiliations:** 1 Service de Radiothérapie, CHU Hassan II Fès, Maroc

**Keywords:** ARTKEYWORDS

## Abstract

In this paper, we report a case of primary squamous cell carcinoma of the endometrium (PSCCE) with a literature review. A 64-year-old woman was admitted because of postmenopausal bleeding. The gynecological exam found bleeding from the endocervix. The pelvic ultrasound objectified uterine regular contours, endometrial thickened was 10 mm, the presence of an intra cavitary lesion measuring 56/70 mm. The diagnostic hysteroscopy revealed a whitish appearance taking all the uterine cavity making evoke a tumor of the endometrium. Pelvic MRI showed a tumor limited to the uterine corpus endometrium (invasion by more than 50% of the myometrium) without invasion of the cervix. Radical hysterectomy, bilateral salpingo-oophorectomy, and lymph nodes dissection were performed. Grossly, the endometrial carcinoma was polypoid tumor occupying the entire uterine cavity. Histologically, the diagnosis of SCC was retained. No adenocarcinoma element was recognized. Neither squamous metaplasia nor dysplasia was recognized. No ectopic cervical tissue was found. The SCC was found to invade into deeper one half of the myometrium. No tumor cells were seen in other sites including the cervix, ovaries, parameters, and lymph nodes. The patients was FIGO 2009 stage IB (pT1B, N0), and was treated with adjuvant radiation. The patient had a disease progression in the pelvis 3 months after the irradiation. We reported a case of PSCCE which can help to enrich the literature for the treatment and prognosis of this disease.

## Introduction

Pure primary endometrial squamous cell carcinoma (PESCC) are extremely rare, accounting for 1 % of all malignancies of the corpus uteri. Since the first report published by Gebhard in 1892, only few cases of PESCC have been published. Here, we report a case of PESCC.

## Patient and observation

**Figure 1:**
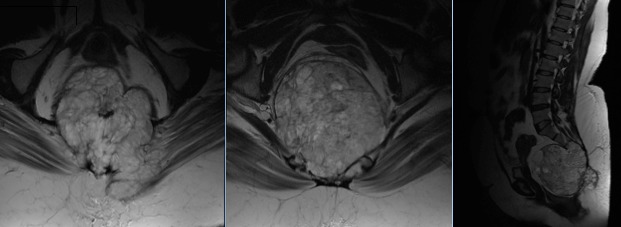
magnetic resonance imaging: large heterogeneous contrast-enhanced presacral mass with irregular contours, pushing the rectum, occupating the canal after lysis of the coccoyx, and involving the gluteal muscles

A 64-year-old woman, Gravida 5 Para 5, was admitted because of postmenopausal bleeding. The gynecological exam found bleeding from the endocervix, uterus had normal size without mass or laterouterine sensitivity. The pelvic ultrasound objectified uterine regular contours, mesuring 103/70 mm, endometrial thickened was 10 mm, the presence of an intra cavitary vascular Doppler lesion mesuring 56/70 mm. The diagnostic hysteroscopy revealed a whitish appearance taking all the uterine cavity making evoke a tumor of the endometrium. Pelvic MRI showed a tumor limited to the uterine corpus endometrium (invasion by more than 50% of the myometrium) without invasion of the cervix. Radical hysterectomy, bilateral salpingo-oophorectomy, and lymph nodes dissection were performed. Grossly, the endometrial carcinoma was polypoid tumor occupying the entire uterine cavity. Histologically, the diagnosis of SCC was retained (Figure 1). No adenocarcinoma element was recognized. Neither squamous metaplasia nor dysplasia was recognized. No ectopic cervical tissue was found. The SCC was found to invade into deeper one half of the myometrium. No tumor cells were seen in other sites including the cervix, ovaries, parametres, and lymph nodes. The patients was FIGO 200 stage IB (pT1B, N0), and was treated with adjuvant radiation. The patient had a disease progression in the pelvis 3 months after the irradiation.

**Figure 2:**
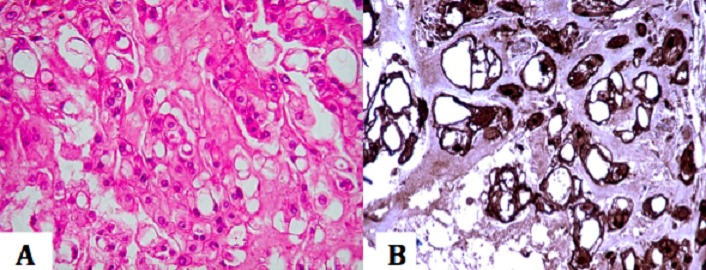
(A) high power view showing, within lobules two populations of cells, small round cells arranged in cords and trabeculae with eosinophilic cytoplasm and round nuclei, second types of cells show vacuolization of cytoplasm giving bubbly appearance known as physaliphorous cells; (B) tumour cells positive for s100

## Discussion

Pure primary endometrial squamous cell carcinoma (PESCC) are extremely rare, accounting for <1 % of all malignancies of the corpus uteri [1]. In the literature, fewer than 100 cases were reported since the first report in 1892 by Gebhard [2]. Diagnosis of PESCC is based on Fluhmann criteria (1928). Briefly, it is mandatory to exclude: cervical carcinoma involving the endometrium, coexistent endometrial adenocarcinoma, and contiguity between the endometrial cancer and the squamous cervix epithelium [3]. The etiopathogenesis of PSCCE is still unknown because of its rarity [4]. Accurate revision of the literature revealed that diverse and controversial hypotheses were suggested by some researchers to clarify causes and pathogenetic mechanisms responsible for PSCCE. In 1993, Horn and Bilek [2] suggested that this malignancy could be the result of a bidirectional differentiation of pluripotent endometrial precursor cells. In 1995, Yamamoto *et al*. [5] in a case report emphasized that PSCCE may arise from heterotropic cervical tissue. More recently, some authors probed to establish if PSCCE could be correlated to human papilloma virus (HPV) infection. The results of these studies are controversial, too. Some authors, in fact, did not detected HPV in cases of PSCCE by in situ hybridization and thus concluded that HPV infection may not be a carcinogenic factor in the development of this neoplasm [6]. Kataoka *et al*. [7] by polymerase chain reaction (PCR) instead demonstrated the presence of human papilloma virus (HPV) type 31 and the absence of mutation of tumor suppressor gene p53.

The clinical features of PSCCE were reported by Goodman *et al*. [8], who found eight cases of this rare neoplasm in a review of 1182 patients treated for endometrial cancer at the Massachusetts General Hospital and 56 cases described previously in literature. The neoplasm occurred in menopausal and postmenopausal women. The factors predisposing to the development of PSCCE included pyometra, pelvic radiation, estrogen deficiency, and estrogen excess [8]. The main clinical manifestations of PSCCE were postmenopausal bleeding, vaginal discharge, pain, weight loss, and pelvic mass. The distant metastases were observed in urethral meatus, vaginal orifice, peritoneal surface, lung, liver, and brain [8]. The average age of women with ESCC, 67 years, is older than the 60 years for patients with corpus carcinoma in general [8]. Molecular alterations in PSCCE leading to cell cycle dysregulation are incompletely investigated. Bures *et al*. [9] reported in a study of 5 cases of PSCCE investigating molecular alterations leading to cell cycle dysregulation, that the PSCCE has molecular alterations involving the pRB-Cyclin D1-CDK4/6-p16 pathway, and pTEN. (Bures) And In contrast to the type I Endometrioid adenocarcinoma of the endometrium, PSCCE is not hormonally sensitive, suggesting a unique pathogenesis [9]. Given the rarity of this condition, there is no consensus for the best way to manage these patients with PSCCE [10]. Many different treatment options have been reported in the literature: surgical resection with or without adjuvant chemotherapy/radiotherapy [10]. Survival data of patients affected by PESCC are scarce and controversial. The few available data suggest that these malignancies have a mixed behavior resembling both endometrial and cervical cancer [1]. For this reason women with early-stage disease have a favorable prognosis, whereas in case of locally advanced cancer, survival is generally poor [1].

## Conclusion

Pure primary endometrial squamous cell carcinoma (PESCC) is an extremely rare malignancy of the corpus uteri. Diagnosis of this rare entity is based on careful pathologic review of the hysterectomy specimen. The underlying etiology or inciting factors leading to this condition have yet to be determined. More studies are needed to address the concern about the extension of primary surgical treatment and the efficacy of adjuvant therapy in this disease.

## Competing interests

The authors declare no competing interests.
